# Non-Alcoholic Fatty Liver Disease and Echocardiographic Parameters of Left Ventricular Diastolic Function: A Systematic Review and Meta-Analysis

**DOI:** 10.3390/ijms241814292

**Published:** 2023-09-19

**Authors:** Athina Goliopoulou, Panagiotis Theofilis, Evangelos Oikonomou, Artemis Anastasiou, Panteleimon Pantelidis, Maria Ioanna Gounaridi, Georgios E. Zakynthinos, Ourania Katsarou, Eva Kassi, Vaia Lambadiari, Dimitris Tousoulis, Manolis Vavuranakis, Gerasimos Siasos

**Affiliations:** 1 3rd Department of Cardiology, Sotiria Chest Disease Hospital, Medical School, National and Kapodistrian University of Athens, 11527 Athens, Greecepan.g.pantelidis@gmail.com (P.P.);; 2 1st Department of Cardiology, Hippokration General Hospital, Medical School, National and Kapodistrian University of Athens, 11527 Athens, Greece; 3 Department of Biological Chemistry, Medical School, National and Kapodistrian University of Athens, 11527 Athens, Greece; ekassi@med.uoa.gr; 4 2nd Department of Internal Medicine, Attikon University Hospital, National and Kapodistrian University of Athens, Medical School, 12462 Athens, Greece

**Keywords:** non-alcoholic fatty liver disease, diastolic dysfunction, left atrial volume, left ventricular mass, heart failure with preserved ejection fraction

## Abstract

The cardiovascular implications of non-alcoholic fatty liver disease (NAFLD) have been associated with heart failure with preserved ejection fraction (HFpEF). The purpose of this review was to conduct a bibliographic search regarding the correlation between NAFLD and the echocardiographic parameters of left ventricular diastolic function. A systematic literature search was conducted in PubMed and Embase for original research data reporting on the association of NAFLD with diastolic function markers [E/e′, left atrial volume index (LAVi), left ventricular mass index (LVMi)]. Meta-analysis was performed using the meta and dmetar packages in R studio v.1.4.1106, with *p* < 0.05 values being considered significant. Results are expressed as the standardized mean difference (SMD) for continuous variables and as the odds ratio (OR) for categorical variables, with respective 95% confidence intervals (CI). Heterogeneity between studies was expressed with index Ι^2^. From the preliminary search, 2619 articles were found from which 31 studies were included in the final statistical analysis. The meta-analysis of 8 studies which reported on the prevalence of diastolic dysfunction showed that it was increased in patients with NAFLD (OR: 2.07, 95% CI 1.24–3.44 with *p* = 0.01, I^2^: 80% with *p* < 0.01). The meta-analysis of 21 studies showed significantly higher E/e′ in NAFLD patients (SMD 1.02, 95% CI 0.43–1.61 with *p* < 0.001, I^2^: 97% with *p* < 0.001). Individuals with NAFLD had increased LAVi (SMD: 0.87, 95% CI 0.38–1.37 with *p* < 0.001, I^2^: 96% with *p* < 0.001) and LVMi (SMD: 0.89, 95% CI 0.31–1.48 with *p* = 0.003, I^2^: 100% with *p* < 0.001). To conclude, in the meta-analysis of 31 observational studies, NAFLD patients were found to have affected left ventricular diastolic function, supporting the hypothesis of NAFLD being associated with HFpEF.

## 1. Introduction

In recent decades, lifestyle changes and socio-economic parameters, such as the aging of the population and poorer dietary habits, have led to an increase in diseases such as type II diabetes mellitus (DM), obesity, and metabolic syndrome [1]. Part of this spectrum is non-alcoholic fatty liver disease (NAFLD), an entity which includes liver diseases with hepatocyte inflammation and fibrosis [2]. An increasing number of individuals are affected by the disease, which is usually part of a broader metabolic situation that affects multiple organ systems [3].

Heart failure is an epidemic with an increasing number of affected individuals. It is a syndrome that affects a wide spectrum of patients since novel drugs and therapeutic strategies have expanded the lifespan of patients with ischemic heart disease, arrythmias, valvulopathies, cardiomyopathies, and other potential causes of heart failure [4]. Among the estimated 6.5 million patients with heart failure in the United States in 2020, approximately half of them have preserved ejection fraction or were otherwise of the “diastolic” phenotype [5]. Heart failure with preserved ejection fraction (HFpEF) is emerging as a syndrome with special characteristics regarding both patient symptoms and signs as well as echocardiographic parameters, clinical course, and prognosis. Various markers have been examined previously, with the E/e′ ratio serving as a means of non-invasive estimation of left ventricular end-diastolic pressure [6]. Additionally, surrogate structural markers such as left atrial volume index (LAVi) and left ventricular mass index (LVMi) have been implicated in diastolic dysfunction [6,7].

Thus far, several studies have highlighted the connection between NAFLD and HFpEF, following the co-existence of overweight and obesity in both of these clinical syndromes [8,9]. Moreover, both NAFLD and HFpEF may share a common pathophysiologic background. Endothelial dysfunction, systemic inflammation, and inflammatory cytokines are found elevated in both entities [10,11,12,13]. Moreover, alteration of glycose and fatty acid metabolism under NAFLD impairs glucose metabolism by the myocardium, leading to energy deficiency and the potentiation of HFpEF [14,15]. However, the clinical correlation between these issues remains understated, and vigilance among clinicians in searching for the possible diagnosis of one entity in the presence of the other remains low.

In this systematic review and meta-analysis, we aim to summarize the existing evidence supporting the association of NAFLD with left ventricular diastolic dysfunction and with the possible HFpEF phenotype. Moreover, we evaluated the structural parameters of the left ventricle indicative of diastolic dysfunction (LAVi, LVMi) in the studied population.

## 2. Materials and Methods

### 2.1. Literature Search Strategy

This systematic review was performed in accordance with the Preferred Reporting Items for Systematic Review and Meta-Analysis Protocols (PRISMA) statement (Table A1 in Appendix A) [16]. The protocol was registered in PROSPERO (ID: CRD42023422697). We conducted a systematic search in the PubMed and Embase search engines up to November 2022 for original research data reporting on the correlation between NAFLD and HFpEF or left ventricular diastolic dysfunction. Two researchers (A.G. and E.O.) independently read and examined the articles regarding their eligibility based on the predefined criteria. Discrepancies among authors were settled through recurrent reviewing in order to reach a consensus.

The details of the queries per database can be found in the Table A2 in Appendix A.

### 2.2. Study Selection Criteria and Data Extraction

Every original observational study (either cohort, cross-sectional, or case–control) published until November 2022 aligning with the PECO framework—(i) participants: general population or specific population groups (adolescents; DM); (ii) exposure: non-alcoholic fatty liver disease; (iii) comparator: absence of non-alcoholic fatty liver disease; (iv) outcomes: HFpEF or left ventricular diastolic dysfunction—was included in our systematic review. The studies which were excluded from the review and meta-analysis were among the following categories: reviews, systematic reviews, meta-analyses, editorials, letters to the editor, and case reports. Non-English publications were also excluded.

From each study, we extracted data regarding the first author’s name, the study type, the sample size, the demographics and other characteristics, the presence of NAFLD, and the indices of left ventricular diastolic function (i.e., prevalence of left ventricular diastolic dysfunction, the E-to-e prime ratio, LAVi, and LVMi).

### 2.3. Quality Assessment and Publication Bias

Quality and risk of bias assessments regarding the 31 studies which ultimately met the eligibility criteria for the meta-analysis were conducted according to the Newcastle–Ottawa Quality Assessment Scale (NOS) criteria adapted for case–control, cross-sectional, and cohort studies regarding study groups’ selection, groups’ comparability, and the determination of either exposure or outcome of interest [17] (see Table A3 in Appendix A).

### 2.4. Statistical Analysis

A meta-analysis was performed to assess the association between NAFLD and diastolic dysfunction prevalence and echocardiographic markers (E/e′, LAVi, and LVMi). I^2^ was chosen as the measure of between-studies heterogeneity, with values over 50% denoting statistical heterogeneity. Effect sizes were pooled via a random-effect model, and the results were presented as the standardized mean difference (SMD) with 95% confidence intervals (CIs) for continuous variables and odds ratio (OR) with 95% CI for categorical variables. Correction for small-sample bias was also applied with the use of Hedge’s g. Moreover, we conducted meta-regressions in order to investigate the effect of age and female sex on the left ventricular diastolic dysfunction prevalence and E/e′ ratio. Prespecified subgroup analyses were also performed according to the included study population and method of NAFLD diagnosis. Sensitivity analyses were performed using the leave-one-out (LOO) approach. Updated meta-analyses were also conducted to assess the associations mentioned above after the exclusion of influential studies. Finally, we examined the existence of potential publication bias through funnel plot inspection and Egger’s test. *p* values less than 0.05 were considered statistically significant. All meta-analyses were performed using the meta and dmetar packages in R studio v.1.4.1106.

## 3. Results

### 3.1. Search Results and Studies Selection

**Figure 1:**
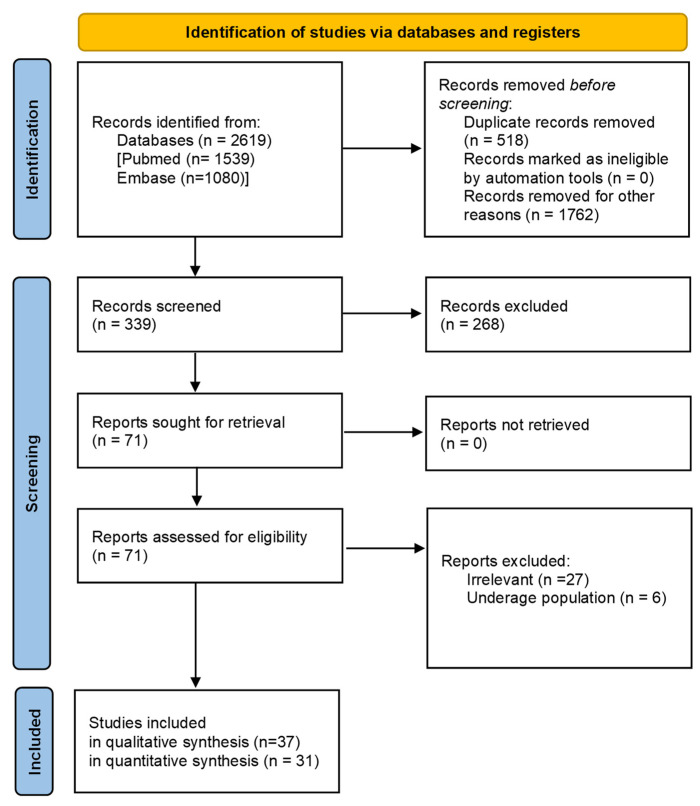
PRISMA flow-chart of the study selection process.

From the initial search, 2619 studies were identified. Ultimately, 38 of them were included in the systematic review [8,18,19,20,21,22,23,24,25,26,27,28,29,30,31,32,33,34,35,36,37,38,39,40,41,42,43,44,45,46,47,48,49,50,51,52,53,54]. Furthermore, 31 of them were found eligible for meta-analysis (Figure 1), since 7 of the studies included in the systematic review did not have the required quantitative data.

From the 31 studies included in the meta-analysis, data on 906,650 subjects with information on NAFLD status and left ventricular diastolic function were meta-analyzed. The main characteristics of the included studies are displayed in Table 1. The mean/median age of the participants in the studies ranged from 31.5 to 68.4. The sample weighted average female-to-male ratio was 1.30, ranging from 0.31 to 3.08.

From the 38 studies, 21 (55%) were cross-sectional, and 12 (32%) were case–control. From the remaining four studies, two were retrospective (5%), and one was prospective observational (3%). The latter study was not included in the meta-analysis since there were no data on the non-NAFLD population. Finally, one study with 1827 participants was a prospective cohort and was included in the meta-analysis.

In most of the studies (16/36), the diagnosis of NAFLD was based on liver ultrasonography. The diagnosis was based on liver biopsy in four studies, and abdominal computed tomography was used for the diagnosis in another five studies. Elastography was used for the diagnosis of NAFLD in three studies. In the rest of the studies, the diagnosis of NAFLD was based on magnetic resonance data, international classification of disease codes, or scores calculating the probability of NAFLD. Interestingly, from the studies included in the meta-analysis, eight of them provided data for NAFLD only for subjects with DM.

### 3.2. Quality Evaluation

Overall, the quality of the studies included in the meta-analysis was high for both cohort and case–control studies, and only one cohort study and one case–control study were found to be of moderate quality with increased risk of bias. Please see Supplementary Materials online, Table A3 in Appendix A) with a detailed report of the NOS quality assessment results.

Despite the adequate quality of the studies included in the meta-analysis, significant heterogeneity exists between the studies, mainly regarding the diagnosis and definition of NAFLD with different tests, diagnostic modalities, or scores. Moreover, heterogeneity was also observed in the selection of the population, with some studies having included only subjects with DM, while most of the studies had selected the general population for analysis. Another source of heterogeneity worth mentioning is the different outcome reported in most studies, since most of them report the value of the E/e’ ratio, and only a few studies evaluate the presence of diastolic dysfunction. Despite the heterogeneity, the results are consistent with a correlation of NAFLD with diastolic dysfunction or surrogate markers of increased left ventricular end diastolic pressure. Heterogeneity and possible misinterpretation of the results may be further worsened by the impact of BMI. Fortunately, most studies provided these data, allowing for the re-evaluation of this association based on more normalized data. Furthermore, only a limited number of studies provided data on the systolic performance of the left ventricle, while there is a lack of data on the true incidence of HFpEF.

### 3.3. Quantitative Synthesis

**Figure 2:**
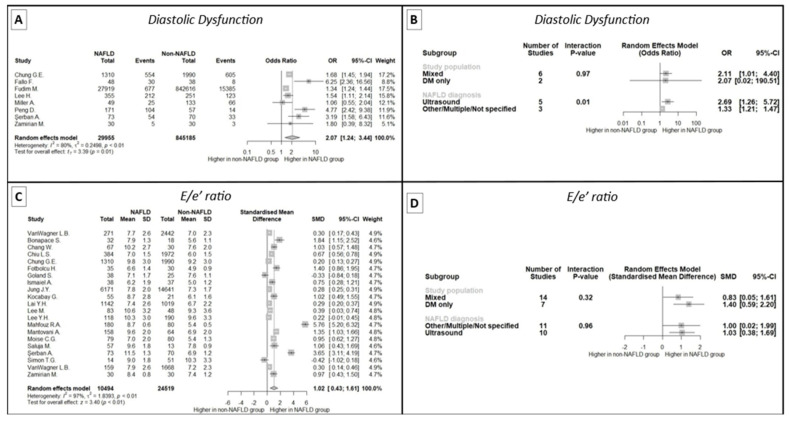
Meta-analysis findings of diastolic dysfunction and E/e′ in patients with and without NAFLD. (**A**): Forest plot of the meta-analysis of eight studies demonstrating a significantly higher prevalence of diastolic dysfunction in patients with NAFLD. (**B**): Subgroup analysis of studies assessing diastolic dysfunction in patients with and without NAFLD, indicating the impact of ultrasonographic diagnosis of NAFLD on the increased prevalence of diastolic dysfunction. (**C**): Forest plot of the meta-analysis of 21 studies demonstrating significantly higher E/e′ in patients with NAFLD. (**D**): Subgroup analysis of studies assessing E/e′ in patients with and without NAFLD, showing no effect of study population or NAFLD diagnosis method. NAFLD: non-alcoholic fatty liver disease, SD: standard deviation, SMD: standardized mean difference, CI: confidence interval, DM: diabetes mellitus.

In a meta-analysis of eight studies that reported the prevalence of diastolic dysfunction, a greater occurrence was noted in NAFLD patients compared to the control group (OR: 2.07, 95% CI 1.24 to 3.44, *p* = 0.01) (Figure 2A). The results remained unaffected after the LOO sensitivity analysis (see Supplementary Material online, Figure S1). However, significant between-study heterogeneity was observed (I^2^: 80%, *p* < 0.01). Interestingly, we found that the prevalence of diastolic dysfunction in NAFLD patients was higher in studies defining NAFLD based solely on liver ultrasonography (Figure 2B). After meta-regressing eight studies, age and sex were not considered significant predictors of the diastolic dysfunction prevalence between NAFLD and non-NAFLD patients, explaining R^2^ = 47.98% of the between-study heterogeneity.

When examining the E/e′ ratio, the meta-analysis of 21 studies displayed a significantly higher E/e′ ratio in patients with NAFLD (SMD 1.02, 95% CI 0.43 to 1.61, *p* < 0.001) (Figure 2C), even after the exclusion of any single study (see Supplementary Material online, Figure S2). Significant between-study heterogeneity was also present (I^2^: 97%, *p* < 0.001), even after exclusion of the outlying studies (see Table A4 in Appendix A). Funnel plot inspection and Egger’s regression test (intercept: 4.06, 95% CI: 1.37 to 6.76, *p* = 0.008) were indicative of publication bias (see Supplementary Material online, Figure S3). No evidence of age and sex interactions were documented in the meta-regression, while subgroup analysis for the method of NAFLD diagnosis and study population according to DM status were unremarkable (Figure 2D).

**Figure 3:**
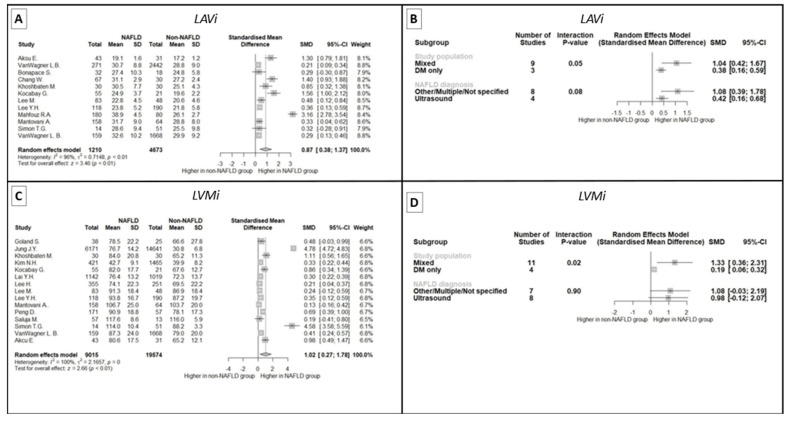
Meta-analysis findings of LAVi and LVMi in patients with and without NAFLD. (**A**): Forest plot of the meta-analysis of 12 studies, demonstrating a significantly higher LAVi in patients with NAFLD. (**B**): Subgroup analysis of studies assessing LAVi in patients with and without NAFLD, showing no effect of NAFLD diagnosis method but a marginal association of diabetic study population with lower effect size. (**C**): Forest plot of the meta-analysis of 20 studies, demonstrating a significantly higher LVMi in patients with NAFLD. (**D**): Subgroup analysis of studies assessing LVMi in patients with and without NAFLD, showing no effect of NAFLD diagnosis method but a significant association of diabetic study population with lower effect size. LAVi: left atrial volume index, LVMi: left ventricular mass index, NAFLD: non-alcoholic fatty liver disease, SD: standard deviation, SMD: standardized mean difference, CI: confidence interval, DM: diabetes mellitus.

LAVi is another index of diastolic function that appears to be affected in the presence of NAFLD, as shown in the meta-analysis of 12 studies (SMD: 0.87, 95% CI 0.38 to 1.37, *p* < 0.001) despite the significant between-study heterogeneity (I^2^: 96%, *p* < 0.001) (Figure 3A). LOO sensitivity analysis did not demonstrate significant changes in effect size even after the exclusion of any single study (see Supplementary Material online, Figure S4). No evidence of publication bias was recorded (intercept 4.98, 95% CI 0.26 to 9.6, *p* = 0.07) (see Supplementary Material online, Figure S5). Interestingly, there was a trend towards lesser differences in LAVi according to the presence of NAFLD in studies handling only patients with DM (Figure 3B).

Moving to LVMi, the meta-analysis of 20 studies highlighted augmented left ventricular mass in subjects with NAFLD (SMD: 0.89, 95% CI 0.31 to 1.48, *p* = 0.003) (Figure 3C), with significant between-study heterogeneity even after the exclusion of influential studies (see Table A3 in Appendix A). As with LAVi, studies including only individuals with DM had non-significant differences in LVMi according to NAFLD (Figure 3D). No publication bias was observed by funnel plot inspection or Egger’s regression test (intercept: −17.22, 95% CI −33.95 to −0.49, *p* = 0.06) (see Supplementary Material online, Figure S6).

## 4. Discussion

This systematic review and meta-analysis of cross-sectional, cohort, and case–control studies investigated the correlation between NAFLD and left ventricular diastolic dysfunction. The present analysis of 31 studies examined the data regarding the echocardiographic parameters of left ventricular diastolic dysfunction and related structural indices in patients with NAFLD and control groups with normal liver function, including a total of 40,760 patients with NAFLD and 869,367 non-NAFLD controls. From this meta-analysis, increased prevalence of left ventricular diastolic dysfunction was found in NAFLD patients compared to the control groups.

NAFLD is a chronic liver disease that results from excessive fat accumulation inside the hepatocytes due to factors other than alcohol consumption [55]. The term encompasses a wide spectrum of liver diseases with ranging severity, from simple steatosis to steatohepatitis leading to fibrosis and ultimately cirrhosis. Hepatocyte fat deposition triggers inflammatory pathways and cellular injury—so-called “ballooning”. The disease is potentially lethal but runs an asymptomatic course over the first years or even decades, misleading patients into lesser attention and delayed lifestyle modifications [56]. NAFLD diagnosis is validated by either radiological liver ultrasound, computed tomography, or magnetic resonance or histologic biopsy findings.

NAFLD pathogenesis is a multifactorial process including cardiovascular diseases; metabolic factors, such as high-fat diet and low levels of physical activity; and genetic polymorphisms. An imbalance in lipid and glucose metabolism is thought to be the cornerstone of NAFLD pathology [3]. Metabolic syndrome and especially type II DM are known to affect lipid metabolism as well as gut microbiota, which in turn contribute to NAFLD pathogenesis via the gut–liver axis. Insulin resistance, present in many patients with inappropriate lipid metabolism, leads to decreased levels of adiponectin, leptin, and other adipocytokines, and furthermore, results in liver free fatty acid intracellular transformation into triglycerides [9]. Alterations in glucose metabolism lead to endoplasmic reticulum stress, inflammation, and increased oxidative stress. Several adipokine-associated molecules, such as tumor necrosis factor-alpha, interleukin-1β, and interleukin-6, have been linked to increased insulin resistance, inflammation, and fat accumulation. The first gene which was identified to participate in NAFLD pathogenesis was patatin-like phospholipase domain-containing protein 3 (PNPLA 3), followed by the also-significant transmembrane 6 superfamily member 2 (TM6SF2) and other genetic variants [56]. Epigenetic alterations also play an important role via changes to the intrahepatic microRNA (miRNA). MiRNAs 122 and 192 have been studied so far. Their levels were found to be upregulated in the serum of patients with hepatic steatosis, and they have been associated with hepatic fatty acid oxidation, liver inflammation, and steatosis in animal models [56].

NAFLD has been associated with cardiovascular disease, and several studies so far have attempted to examine the correlation between fatty liver disease and heart failure with systolic or diastolic left ventricular dysfunction. Most importantly, HFpEF and diastolic dysfunction are often part of a joint patient phenotype with fatty liver disease, and studies have tried to specify the relationship between the two entities as well as possible causal explanations of their co-existence. In this systematic review and meta-analysis, we aimed at investigating studies which compared patients with NAFLD to controls with normal liver function, regarding left ventricular diastolic function, as assessed by echocardiographic parameters.

Among the studies included in the meta-analysis, eight studies clearly examined the existence of diastolic dysfunction. In our meta-analysis, increased prevalence of diastolic dysfunction was found in NAFLD patients in comparison to the control groups. The results were not altered after LOO sensitivity analysis, but the limitation of between-study heterogeneity should be noted. This difference in diastolic function between groups was more pronounced in studies which used liver ultrasonography to define NAFLD. Following meta-regression of the eight studies, patient age and sex were not found to be important predictors of diastolic dysfunction in the NAFLD and non-NAFLD groups.

A meta-analysis of 21 studies was conducted regarding the E/e’ ratio as a cornerstone of echocardiographic evaluation of left ventricular diastolic dysfunction. The ratio of the early diastolic velocity of mitral inflow (E) to the early diastolic velocity of mitral annular motion (e′) is a studied parameter that best reflects left ventricular filling pressures and increases in patients with increased pulmonary capillary wedge pressure. It is used in clinical practice as a measure of diastolic dysfunction severity [4]. In this meta-analysis, E/e′ was increased in the NAFLD population compared to the control groups, while, interestingly, demographic parameters like age and sex did not affect the results. Subgroup analysis regarding patients with DM as well as comparison between diagnostic modalities for NAFLD, did not show significant differences. These findings reflect a direct relationship between fatty liver disease and diastolic dysfunction, unaffected by other metabolic diseases or older age.

LAVi is an echocardiographic feature characteristic of cardiac structural alterations in patients with left ventricular diastolic dysfunction. Different phases of left atrial function are affected by different types of left ventricular dysfunction. Left ventricular systolic dysfunction usually affects left atrial relaxation which occurs during systole, while left ventricular diastolic dysfunction with increased wall stiffness, impaired relaxation, and increased end-diastolic pressures mostly affects left atrial conduit and booster phases during diastole. Increased left atrial size is a sign of left ventricular diastolic dysfunction and is especially reflective of its chronicity and severity, since atrial size increase occurs gradually over time. In the relevant meta-analysis of 12 studies, LAVi was shown to be increased in NAFLD patient groups compared to controls. It is notable that studies which solely included patients with DM exhibited a trend towards a lesser difference in LAVi.

LVMi is another indicator of diastolic dysfunction. It is increased in patients with impaired left ventricular relaxation and increased filling pressures. LVMi was measured in 20 of the reviewed studies. In our meta-analysis of these 20 studies, LVMi was increased in NAFLD patients. As with LAVi, studies comparing NAFLD and non-NAFLD patient groups with DM did not have significant differences in LVMi.

The results of this meta-analysis have various clinical implications. The establishment of the association of NAFLD with HFpEF and left ventricular diastolic dysfunction is of critical clinical significance since it could prompt physicians to look for fatty liver disease in the presence of HFpEF and vice versa. Such an approach could lead to a much earlier diagnosis of these diseases, which are asymptomatic during the early years, and a more effective therapeutic approach. This correlation highlights the importance of treating patients holistically, keeping in mind that there is significant interaction between the heart and other organs, especially liver function.

The pathophysiology remains a field of ongoing research. NAFLD is characterized by the accumulation of excess fat in the liver, leading to insulin resistance, chronic inflammation, and oxidative stress. These systemic effects extend beyond the liver, affecting various organ systems, including the cardiovascular system. In NAFLD, the chronic inflammation and release of pro-inflammatory cytokines can induce endothelial dysfunction, impairing the relaxation and compliance of cardiac blood vessels [57]. Moreover, insulin resistance and metabolic dysregulation, common in NAFLD, can lead to myocardial fibrosis and hypertrophy, which further compromise diastolic function [57]. Additionally, adipokines and hepatokines produced by the fatty liver can contribute to systemic inflammation and oxidative stress, exacerbating myocardial dysfunction [57]. This intricate web of metabolic, inflammatory, and vascular disturbances collectively contributes to the higher prevalence of diastolic dysfunction and HFpEF in NAFLD patients.

While there have already been studies that demonstrate a correlation between NAFLD and left ventricular function, this meta-analysis is the first to encompass multiple echocardiographic parameters of diastolic function, including structural alterations such as left atrial enlargement. Moreover, there was a special examination of patients with type II diabetes mellitus, a disease known to be of increased prevalence among NAFLD patients. Previous meta-analyses have also provided similar findings [58,59], further documenting the tight relationship between the two entities.

The most significant limitation of our study was the heterogeneity between the studies included in the meta-analysis. The results remained unaltered even after LOO sensitivity analysis and the exclusion of outlying studies. Another factor which could be a limitation is the use of different means for NAFLD diagnosis. Among the studies which were compared, some used liver ultrasound for the establishment of NAFLD diagnosis, while others used computed tomography or magnetic resonance and others liver biopsy or a combination of methods. Due to the observational design of the studies which were included in the review, causality between NAFLD and diastolic or systolic dysfunction of the left ventricle can neither be confirmed nor denied. Finally, the inclusion of only one prospective study in the meta-analysis remains a limitation.

## 5. Conclusions

This systematic review and meta-analysis summarizes the interplay between NAFLD and left ventricular diastolic dysfunction, as evidenced by surrogate echocardiographic markers of elevated left ventricular end-diastolic pressure (E/e′) or structural parameters indicative of diastolic dysfunction (LAVi, LVMi). According to our results, NAFLD patients are associated with an increased risk of developing left ventricular diastolic dysfunction. This correlation is clinically significant, as it raises clinicians’ sensitivity towards a holistic patient approach, leading to possible early diagnosis of one disease in the presence of the other and a simultaneous course of treatment. Additional studies are needed in order to further elucidate the pathophysiologic connections of the association between NAFLD and diastolic dysfunction or HFpEF.

## Figures and Tables

**Table 1:** Characteristics of the studies included in the systematic review and meta-analysis.

Study	SR/MA	Sample Size		Age (Years)	Sex (Female) %	Study Type	NAFLD Diagnosis	Key Findings
		Non-NAFLD	NAFLD					
Chiu L.S. et al. [8]	MA	1972	384	52.1 ± 12	51.8	Cross-sectional	Abdominal CT	Framingham Heart Study Third Generation cohort NAFLD patients had lower E/A, e′ and higher E, E/e′, LV mass. BMI was a significant mediator between liver fat and LV diastolic dysfunction parameters.
Aksu E et al. [18]	MA	43	31	32 ± 4	100	Case–control	Liver ultrasonography	Diastolic dysfunction indices were higher in the group of NAFLD compared to non-NAFLD (lateral E/e′ 5.8 ± 1.9 vs. 5.5 ± 2.0, *p* = 0.61, septal E/e′ 7.8 ± 2.2 vs. 6.5 ± 2.0, *p* = 0.01). Moreover, the NAFLD group showed increased parameters of left ventricle hypertrophy, left atrial size, as well as increased inter- and intra- atrial electromechanical delay.
Aparci et al. [19]	MA	102	56	34.0 ± 6.7	34.2	Cross-sectional	Liver ultrasonography	NAFLD patients had lower E/A and significantly greater LA diameter. No information on E/e′ was provided.
VanWagner L.B. et al. [20]	MA	2442	271	50.1 ± 3.6	46.5	Cross-sectional	Abdominal CT	NAFLD patients had higher LVMi, higher E/e’—findings of subclinical cardiac remodeling in systolic as well as diastolic function.
Bonapace S et al. [21]	MA	18	32	64.1 ± 4.8	24	Cross-sectional	Liver ultrasonography	T2DM population Diastolic dysfunction in NAFLD patients Increased LV filling pressures
Cassidy S et al. [22]	MA	19	19	55 ± 15	42	Case–control	^1^H-magnetic resonance spectroscopy of the liver	Adults with NAFLD and T2DM demonstrate concentric remodeling with an elevated eccentricity ratio compared to controls. No data are provided on the ratio of E/e′, with similar LVMi across the two groups.
Chang W. et al. [23]	MA	30	67	47.1 ± 8.9	33	Cross-sectional	Liver ultrasonography	T2DM population. No significant difference among controls and mild NAFLD. LA strain values decreased in severe NAFLD group.
Chung G.E. et al. [24]	MA	1990	1310	54 ± 10.2	36.8	Cross-sectional	Liver ultrasonography	Increased prevalence of LV diastolic dysfunction in NAFLD groups, as defined by E/A, E/e′, septal e′, LA, and LV dimensions. Increased risk of diastolic dysfunction according to fibrosis in non-obese patient group following patient stratification according to BMI.
Fallo F. et al. [25]	MA	38	48	49 ± 10	32	Case–control	Liver ultrasonography	Essential hypertensives patients No data are provided regarding E/e’ ratio. Higher prevalence of diastolic dysfunction (62.5% vs. 21.1%, *p* < 0.001) in NAFLD compared to control subjects, as defined by E/A ratio < 1 and E-wave deceleration time > 220 ms.
Fotbolcu H et al. [26]	MA	30	35	40.3 ± 6.2	41.5	Case–control	Liver ultrasonography	NAFLD patients had lower E/A and e’, increased DT, IVRT, and E/e’—increased LV filling pressures and impaired diastolic function.
Goland S. et al. [27]	MA	25	38	44.8 ± 6.6	24.9	Case–control	Liver biopsy	NAFLD patients had altered LV geometry with pronounced thickening of IVS and PW, lower E/A, and increased DT. However, no significant differences in LV filling pressures (E/e’) were reported.
Ismaiel A. et al. [28]	MA	37	38	42.1 ± 18.8	53.4	Cross-sectional	Liver ultrasonography SteatoTest	MAFLD patients had lower E/A compared to healthy controls and increased LV filling pressures as defined by E/e’.
Jung J.Y. et al. [29]	MA	14,641	6171	39.7 ± 7.6	37.1	Cross-sectional	Liver ultrasonography	Impaired LV relaxation in NAFLD patients, with a correlation between NAFLD severity and degree of LV remodeling and diastolic dysfunction as measured with E/e’, LV mass, LVEDV, E/A, and tissue e’ velocities.
Khoshbaten M et al. [30]	MA	30	30	40 ± 7	40	Case–control	Liver ultrasonography	NAFLD patients had increased LAVi compared to the controls.
Kim NH et al. [31]	MA	1465	421	56.6 ± 7.3	62	Cross-sectional	Computed tomography	4 groups: with and without NAFLD, with and without MetS. No significant differences. The presence of NAFLD in subjects with MetS additively contributed to a subclinical deterioration in LV diastolic function.
Kocabay G et al. [32]	MA	21	55	42.1 ± 7.3	43.1	Cross-sectional	Liver biopsy	LA geometry and functional properties assessed by speckle-tracking echo. NAFLD patients had lower peak strain during atrial and ventricular systole. LA strain during ventricular systole was significantly associated with E, Em, and LAVi values. Atrial deformation parameters did not significantly differ among NAFLD groups according to liver disease severity.
Lai YH et al. [33]	MA	1019	1142	48.1 ± 7.3	36.3	Retrospective cohort	Liver ultrasonography	NAFLD patients with increased fibrosis had significantly elevated E/e’, LA stiffness, decreased e’, and decreased LA strain values, independent of cardiovascular disease risk factors and obesity.
Lee H. et al. [34]	MA	251	355	62.7 ± 5.1	75.5	Cross-sectional	Liver ultrasonography	T2DM population LV diastolic dysfunction prevalence higher in NAFLD group with increased LV mass, LA dimensions, lower E/A ratio, and longer DT.
Lee M et al. [35]	MA	48	83	60	44.3	Cross-sectional	Elastography	T2DM patients NAFLD group had diastolic dysfunction with increased LV filling pressures (E/e’) and LAVi. Higher degree of hepatic fibrosis independently associated with higher E/e’ ratio and decreased myocardial FDG uptake in PET
Lee YH et al. [36]	MA	190	118	57.1	44.9	Cross-sectional	Elastography	NAFLD patients showed increased LV wall thickness, ventricular and atrial volumes, LV diastolic dysfunction as assessed by decreased e’ and increased LV filling pressures (E/e’), and atrial systolic dysfunction with reduced atrial longitudinal strain and increased atrial stiffness
Mahfouz RA et al. [37]	MA	80	180	47.6	46.6	Case–control	Elastography	NAFLD patients had increased LA stiffness index values [as calculated with (E/e’)/LA global PALS ratio], interatrial septum thickness, LAVi and E/e’. Higher AF prevalence in NAFLD group, possibly related to altered LA geometry
Mantovani, A. et al. [38]	MA	64	158	67.4	29.6	Cross-sectional	Liver ultrasonography	T2DM outpatient population NAFLD group had echocardiographic features of diastolic dysfunction; lower e’ and increased E/e’, LVEDP, and LAVi
Miller A et al. [39]	MA	133	49	68.4 ± 12.9	58.6	Cross-sectional	US/MRI/CT/biopsy/ICD-9/10	HFpEF patients 27% met NAFLD criteria with higher rates of NYHA III-IV HF symptoms and diastolic dysfunction grade ≥ 2, increased IVS thickness and LAVi.
Moise CG et al. [40]	MA	80	79	31.5 ± 6.8	38.8	Case–control	Liver ultrasonography	Young (15–45) adult population Hepatic steatosis was associated with lower e’ velocities, higher E/A, E/e’. Concomitant DM did not affect diastolic dysfunction parameters.
Peng D et al. [41]	MA	57	171	47.8 ± 12.1	32	Cross-sectional	Liver ultrasonography or transient elastography	Moderate-to-to severe steatosis patients had higher risks for left ventricle diastolic dysfunction and cardiac remodeling with higher LVMi.
Saluja M et al. [42]	MA	13	57	55.7 ± 10.4	45.1	Cross-sectional	Liver ultrasonography	T2DM population NAFLD patient group had decreased e’ tissue velocities, increased E/e’ ratio, and elevated LVEDP.
Şerban A et al. [43]	MA	70	73	57.5 ± 3.5	28.9	Case–control	Liver ultrasonography	T2DM population NAFLD patient group had lower e’ tissue velocities, higher E/e’, more severe diastolic dysfunction compared to controls.
Simon TG et al. [44]	MA	51	14	48.4 ± 12.3	40.1	retrospective cohort	Liver biopsy	NAFLD patients had diastolic dysfunction echocardiographic parameters such as increased LAVi and LVMi, decreased e’ tissue velocities, E, E/A, and DT.
L. B. VanWagner et al. [45]	MA	1668	159	49.9 ± 3.6	60.6	Prospective cohort	Computed Tomography	From CARDIA study NAFLD patients had increased LAVi, LV mass and impaired LV relaxation with elevated LV filling pressures (higher E/e’).
Zamirian M et al. [46]	MA	30	30	37.6 ± 4.7	48.3	Case–control	Liver biopsy/ ultrasonography	NAFLD patients had altered LV geometry with increased diameters as well as diastolic dysfunction with lower e’ tissue velocities and higher E/e’.
Canada J McN et al. [47]	SR	-	36	54 (48–60)	67	Cross-sectional	Biopsy confirmed	NASH was compared to NAFL. Diastolic function was assessed according to liver fibrosis. E/e’ during exercise increased progressively with increasing fibrosis. NASH was associated with impaired exercise capacity compared to NAFL.
Fudim M et al. [48]	ΜA	842,616	27,919	74.5 ± 7.1	57	Cohort study	International Classification of Diseases	Patients with (versus without) baseline NAFLD had a significantly higher risk of new-onset HF. Among HF subtypes, the association of NAFLD with downstream risk of HF was stronger for HFpEF.
Furuhashi M et al. [49]	SR	-	185	63 ± 14	43	Cross-sectional	Fatty liver index	Elevated fatty liver index is independently associated with LV diastolic dysfunction in a general population without medication.
Makker J et al. [50]	SR	94	64	-	-	Case–control	Computed tomography	Severe NAFLD compared to control was associated with a higher left ventricular mass after normalization for height^2.7^.
Petta S. et al. [51]	SR	-	147	48 ± 12	36	Cross-sectional	Biopsy confirmed	Left ventricular mass, relative wall thickness, and left atrial volume, as well as E/A ratio and diastolic dysfunction were linked to severe liver fibrosis.
Sonaglioni A et al. [52]	SR	-	92	54 ± 11	50	Cross-sectional	Liver stiffness measurement	12.0% of the NAFLD patients were found with normal diastolic filling pattern, 7.6% showed a pseudonormal diastolic filling pattern, and no patient was diagnosed with restrictive filling pattern. Left ventricular filling pressures as expressed by the average E/e’ ratio, were in the “gray zone” of 8 to 13 (average E/e’ ratio 10.0 ± 2.9).
Ybarra J et al. [53]	SR	-	151	38.4 ± 07	76	Cross-sectional	Liver ultrasonography	Increased prevalence of LVH according to ALT levels. Lower E/A ration according to ALT levels.
Yoshihisa A. Et al. [54]	SR		492	69.8 ± 13.9	50.2	Prospective observational	Non-alcoholic fatty liver disease fibrosis score	Patients with HFpEF and NAFLD. Higher NAFLD fibrosis score is associated with higher mortality, and higher BNP levels.

SR: systematic review, MA: meta-analysis, NAFLD: non-alcoholic fatty liver disease, LA: left atrial, LVMi: left ventricular mass index, T2DM: type 2 diabetes mellitus, BMI: body mass index, IVRT: isovolumic relaxation time, DT: deceleration time, IVS: interventricular septum, PW: posterior wall, LVEDV: left ventricular end-diastolic volume, MetS: metabolic syndrome, LAVi: left atrial volume index, HFpEF: heart failure with preserved ejection fraction, NYHA: New York Heart Association, LVEDP: left ventricular end-diastolic pressure, ALT: alanine transaminase, BNP: brain natriuretic peptide.

## Data Availability

The data supporting the results of this study are available upon reasonable request from the corresponding author.

## Supplementary Materials

The following supporting information can be downloaded at: https://www.mdpi.com/article/10.3390/ijms241814292/s1.

## Appendix A

**Table A1:** PRISMA 2020 Checklist of the systematic review and meta-analysis.

Section and Topic	Item #	Checklist Item	Location Where Item Is Reported
**TITLE**			
Title	1	Identify the report as a systematic review.	p. 1
**ABSTRACT**			
Abstract	2	See the PRISMA 2020 for Abstracts checklist.	p. 1
**INTRODUCTION**			
Rationale	3	Describe the rationale for the review in the context of existing knowledge.	p. 2
Objectives	4	Provide an explicit statement of the objective(s) or question(s) the review addresses.	p. 2
**METHODS**			
Eligibility criteria	5	Specify the inclusion and exclusion criteria for the review and how studies were grouped for the syntheses.	p. 2
Information sources	6	Specify all databases, registers, websites, organisations, reference lists and other sources searched or consulted to identify studies. Specify the date when each source was last searched or consulted.	p. 2
Search strategy	7	Present the full search strategies for all databases, registers and websites, including any filters and limits used.	Appendix A1
Selection process	8	Specify the methods used to decide whether a study met the inclusion criteria of the review, including how many reviewers screened each record and each report retrieved, whether they worked independently, and if applicable, details of automation tools used in the process.	p. 2
Data collection process	9	Specify the methods used to collect data from reports, including how many reviewers collected data from each report, whether they worked independently, any processes for obtaining or confirming data from study investigators, and if applicable, details of automation tools used in the process.	p. 2
Data items	10a	List and define all outcomes for which data were sought. Specify whether all results that were compatible with each outcome domain in each study were sought (e.g., for all measures, time points, analyses), and if not, the methods used to decide which results to collect.	p. 2
	10b	List and define all other variables for which data were sought (e.g., participant and intervention characteristics, funding sources). Describe any assumptions made about any missing or unclear information.	p. 2
Study risk of bias assessment	11	Specify the methods used to assess risk of bias in the included studies, including details of the tool(s) used, how many reviewers assessed each study and whether they worked independently, and if applicable, details of automation tools used in the process.	p. 3
Effect measures	12	Specify for each outcome the effect measure(s) (e.g., risk ratio, mean difference) used in the synthesis or presentation of results.	p. 3
Synthesis methods	13a	Describe the processes used to decide which studies were eligible for each synthesis (e.g., tabulating the study intervention characteristics and comparing against the planned groups for each synthesis (item #5)).	p. 2
	13b	Describe any methods required to prepare the data for presentation or synthesis, such as handling of missing summary statistics, or data conversions.	p. 3
	13c	Describe any methods used to tabulate or visually display results of individual studies and syntheses.	p. 3
	13d	Describe any methods used to synthesize results and provide a rationale for the choice(s). If meta-analysis was performed, describe the model(s), method(s) to identify the presence and extent of statistical heterogeneity, and software package(s) used.	p. 3
	13e	Describe any methods used to explore possible causes of heterogeneity among study results (e.g., subgroup analysis, meta-regression).	p. 3
	13f	Describe any sensitivity analyses conducted to assess robustness of the synthesized results.	p. 3
Reporting bias assessment	14	Describe any methods used to assess risk of bias due to missing results in a synthesis (arising from reporting biases).	p. 3
Certainty assessment	15	Describe any methods used to assess certainty (or confidence) in the body of evidence for an outcome.	
**RESULTS**			
Study selection	16a	Describe the results of the search and selection process, from the number of records identified in the search to the number of studies included in the review, ideally using a flow diagram.	p.3–4 Figure 1
	16b	Cite studies that might appear to meet the inclusion criteria, but which were excluded, and explain why they were excluded.	p.3–4 Figure 1
Study characteristics	17	Cite each included study and present its characteristics.	Table 1
Risk of bias in studies	18	Present assessments of risk of bias for each included study.	Appendix A2
Results of individual studies	19	For all outcomes, present, for each study: (a) summary statistics for each group (where appropriate) and (b) an effect estimate and its precision (e.g., confidence/credible interval), ideally using structured tables or plots.	p. 11–13 Figures 2–4
Results of syntheses	20a	For each synthesis, briefly summarise the characteristics and risk of bias among contributing studies.	p. 11–13
	20b	Present results of all statistical syntheses conducted. If meta-analysis was done, present for each the summary estimate and its precision (e.g., confidence/credible interval) and measures of statistical heterogeneity. If comparing groups, describe the direction of the effect.	p. 12–15 Figures 2–4
	20c	Present results of all investigations of possible causes of heterogeneity among study results.	p. 11–13 Figures 2 and 3 Figures S1, S2, S4
	20d	Present results of all sensitivity analyses conducted to assess the robustness of the synthesized results.	p. 11–13 Figure S1, S2, S4
Reporting biases	21	Present assessments of risk of bias due to missing results (arising from reporting biases) for each synthesis assessed.	Appendix A2
Certainty of evidence	22	Present assessments of certainty (or confidence) in the body of evidence for each outcome assessed.	
**DISCUSSION**			
Discussion	23a	Provide a general interpretation of the results in the context of other evidence.	p. 13–16
	23b	Discuss any limitations of the evidence included in the review.	p. 15
	23c	Discuss any limitations of the review processes used.	p. 15
	23d	Discuss implications of the results for practice, policy, and future research.	p. 15
**OTHER INFORMATION**			
Registration and protocol	24a	Provide registration information for the review, including register name and registration number, or state that the review was not registered.	p. 2
	24b	Indicate where the review protocol can be accessed, or state that a protocol was not prepared.	p. 2
	24c	Describe and explain any amendments to information provided at registration or in the protocol.	
Support	25	Describe sources of financial or non-financial support for the review, and the role of the funders or sponsors in the review.	p. 16
Competing interests	26	Declare any competing interests of review authors.	p. 16
Availability of data, code and other materials	27	Report which of the following are publicly available and where they can be found: template data collection forms; data extracted from included studies; data used for all analyses; analytic code; any other materials used in the review.	p. 18

**Table A2:** Search Queries for the literature search of the meta-analysis from the examined databases.

Database	Query
PubMed	(“heart failure with preserved ejection fraction” OR “HFpEF” OR “E/A” OR “E/e” OR “E/e” OR “tissue doppler” OR “peak atrial longitudinal strain” OR “PALS” OR “atrial longitudinal strain rate” OR “left atrial strain” OR “left atrial stiffness” OR “left atrial” OR “left atrial volume” OR “left atrial volume index” OR “LAVi” OR “left atrial diameter” OR “diastolic dysfunction” OR “diastolic heart failure” OR “diastolic impairment” OR “diastolic”) AND (“nonalcoholic fatty liver disease” OR “non-alcoholic fatty liver disease” OR “NAFLD” OR “metabolic dysfunction fatty liver disease” OR “MAFLD” OR “fatty liver” OR “fatty liver disease” OR “steatosis” OR “liver steatosis” OR “hepatic steatosis” OR “steatohepatitis” OR “nonalcoholic steatohepatitis” OR “non-alcoholic steatohepatitis” OR “NASH”)
Embase	(‘heart failure with preserved ejection fraction’ OR HFpEF OR E/A OR E/e OR E/e' OR ‘tissue doppler’ OR ‘peak atrial longitudinal strain’ OR PALS OR ‘atrial longitudinal strain rate’ OR ‘left atrial strain’ OR ‘left atrial stiffness’ OR ‘left atrial’ OR ‘left atrial volume’ OR ‘left atrial volume index’ OR LAVi OR ‘left atrial diameter’ OR ‘diastolic dysfunction’ OR ‘diastolic heart failure’ OR ‘diastolic impairment’ OR diastolic) AND (‘nonalcoholic fatty liver disease’ OR ‘non-alcoholic fatty liver disease’ OR NAFLD OR ‘metabolic dysfunction fatty liver disease’ OR MAFLD OR ‘fatty liver’ OR ‘fatty liver disease’ OR steatosis OR ‘liver steatosis’ OR ‘hepatic steatosis’ OR steatohepatitis OR ‘nonalcoholic steatohepatitis’ OR ‘non-alcoholic steatohepatitis’ OR NASH)

**Table A3:** Quality Assessment Results with the Newcastle–Ottawa Quality Assessment Scale (NOS) Tool of the studies included in the meta-analysis.

ID	Study Design	Selection	Comparability	Exposure/Outcome	Score
Aksu E. et al. 2021 [18]	cross-sectional	***	*	***	7
Aparci M. et al. 2010 [19]	cross-sectional	***	*	***	7
Bonapace S et al. 2012 [21]	cross-sectional	***	**	***	8
Cassidy S. et al. 2015 [22]	case control	****	**	**	8
Chang W. et al. 2019 [23]	cross-sectional	***	*	***	7
Chiu L.S. et al. 2020 [8]	cross-sectional	*****	**	***	9
Chung G.E. et al. 2018 [24]	cross-sectional	****	*	***	8
Fallo F. et al. 2009 [25]	cross-sectional	***	**	***	8
Fotbolcu H et al. 2010 [26]	case-control	***	*	***	7
Fudim M et al. 2021 [48]	retrospective cohort	****	*	***	8
Goland S. et al. 2006 [27]	case-control	****	**	***	9
Ismaiel A. et al 2022 [28]	cross-sectional	***	*	***	7
Jung J.Y. et al 2017 [29]	cross-sectional	****	*	***	8
Khoshbaten M. et al. 2015 [30]	case-control	**	**	***	7
Kim NH et al. 2014 [31]	cross-sectional	*****	*	***	9
Kocabay G et al 2014 [32]	cross-sectional	***	*	***	7
Lai YH et al 2022 [33]	retrospective cohort	****	*	***	8
Lee H et al. 2020 [34]	cross-sectional	****	**	***	9
Lee M et al. 2021 [35]	cross-sectional	****	*	***	8
Lee YH et al., 2018 [36]	cross-sectional	***	*	***	7
Mahfouz RA et al., 2019 [37]	case-control	**	**	**	6
Mantovani, A. et al., 2015 [38]	cross-sectional	****	**	***	9
Miller A et., 2020 [39]	cross-sectional	*****	**	***	9
Moise CG et al., 2021 [40]	case-control	**	**	***	7
Peng D et al. 2022 [41]	cross-sectional	****	**	***	9
Saluja M et al., 2019 [42]	cross-sectional	***	**	***	8
Şerban A et al., 2012 [43]	case-control	***	**	***	8
Simon TG et al., 2017 [44]	retrospective cohort	**	*	**	5
L. B. VanWagner et al., 2020 [45]	prospective	****	**	***	9
VanWagner L.B. et al. 2015	cross-sectional	*****	**	**	8
Zamirian M et al., 2018 [46]	case control	****	**	***	9

*: Number of stars as per quality assessment in each category.

**Table A4:** Updated Meta-Analyses Results after Removal of Outlying Studies.

Marker	Analysis	SMD (95% CI)	P	τ^2^	Ι^2^
**LVMi**	Main Analysis	0.89 (0.31, 1.47)	0.003	1.71	100%
	Outlying studies removed	0.48 (0.29, 0.67)	<0.001	0.12	68%
**LAVi**	Main Analysis	0.87 (0.38, 1.37)	<0.001	0.72	96%
	Outlying studies removed	0.69 (0.38, 1.01)	<0.001	0.21	81%
**E/e’**	Main Analysis	1.02 (0.43, 1.61)	<0.001	1.84	97%
	Outlying studies removed	0.87 (0.62, 1.12)	<0.001	0.17	85%

LVMi: left ventricular mass index, LAVi: left atrial volume index, SMD: standardized mean difference, CI: confidence intervals.
