# Supplementary material for: Non-Alcoholic Fatty Liver Disease and Echocardiographic Parameters of Left Ventricular Diastolic Function: A Systematic Review and Meta-Analysis
Source: Int J Mol Sci. 2023 Sep 19;24(18):14292. doi: 10.3390/ijms241814292 (PMC10532416; doi:10.3390/ijms241814292)
Supplement: Supplementary file 1 [file ijms-24-14292-s001.zip › Supplementary Figure legend.pdf]

**Supplementary Figure S1.** Exclusion of any single study using the leave-one-out method did not alter the results of the meta-analysis concerning diastolic dysfunction in patients with and without NAFLD. The leave-one-out sensitivity analysis ordered by effect size (θ) was performed, with $I^2$ representing between-study heterogeneity and horizontal error bars denoting the 95% confidence intervals.

**Supplementary Figure S2.** Exclusion of any single study using the leave-one-out method did not alter the results of the meta-analysis concerning E/e’ in patients with and without NAFLD. The leave-one-out sensitivity analysis ordered by effect size (θ) was performed, with $I^2$ representing between-study heterogeneity and horizontal error bars denoting the 95% confidence intervals.

**Supplementary Figure S3.** Inspection of the funnel plot of studies assessing E/e’ in patients with and without NAFLD indicates asymmetry, potentially caused by publication bias. Hedges’ g was used as the effect size metric.

**Supplementary Figure S4.** Exclusion of any single study using the leave-one-out method did not alter the results of the meta-analysis concerning LAVi in patients with and without NAFLD. The leave-one-out sensitivity analysis ordered by effect size (θ) was performed, with $I^2$ representing between-study heterogeneity and horizontal error bars denoting the 95% confidence intervals.

**Supplementary Figure S5.** Inspection of the symmetric funnel plot of studies assessing LAVi in patients with and without NAFLD. Hedges’ g was used as the effect size metric.

**Supplementary Figure S6.** Inspection of the symmetric funnel plot of studies assessing LVMi in patients with and without NAFLD. Hedges’ g was used as the effect size metric.
