# Supplementary figures and images for: Non-Alcoholic Fatty Liver Disease and Echocardiographic Parameters of Left Ventricular Diastolic Function: A Systematic Review and Meta-Analysis
Source: Int J Mol Sci. 2023 Sep 19;24(18):14292. doi: 10.3390/ijms241814292 (PMC10532416; doi:10.3390/ijms241814292)

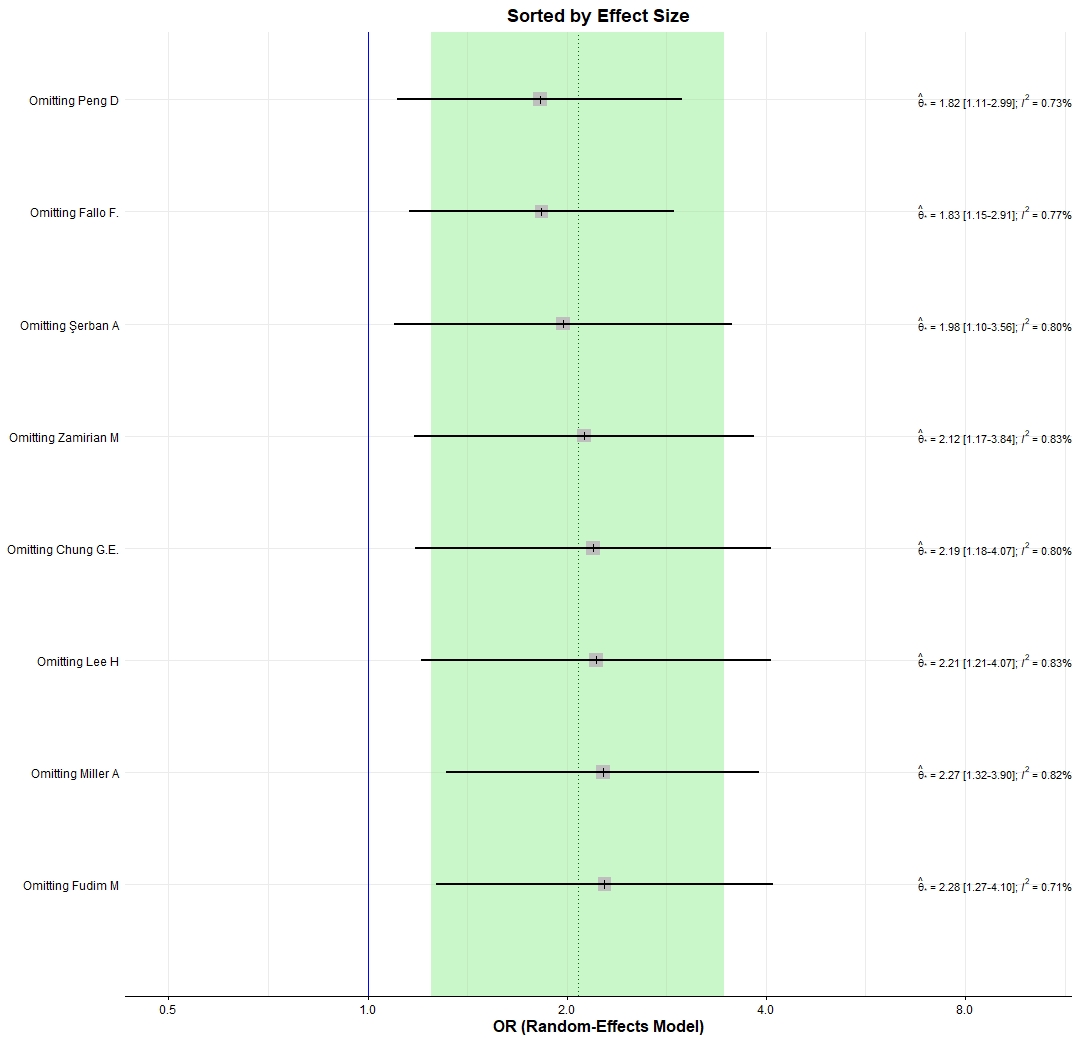

Supplement: Supplementary file 1 [file ijms-24-14292-s001.zip › Supplementary Figure S1.jpeg]

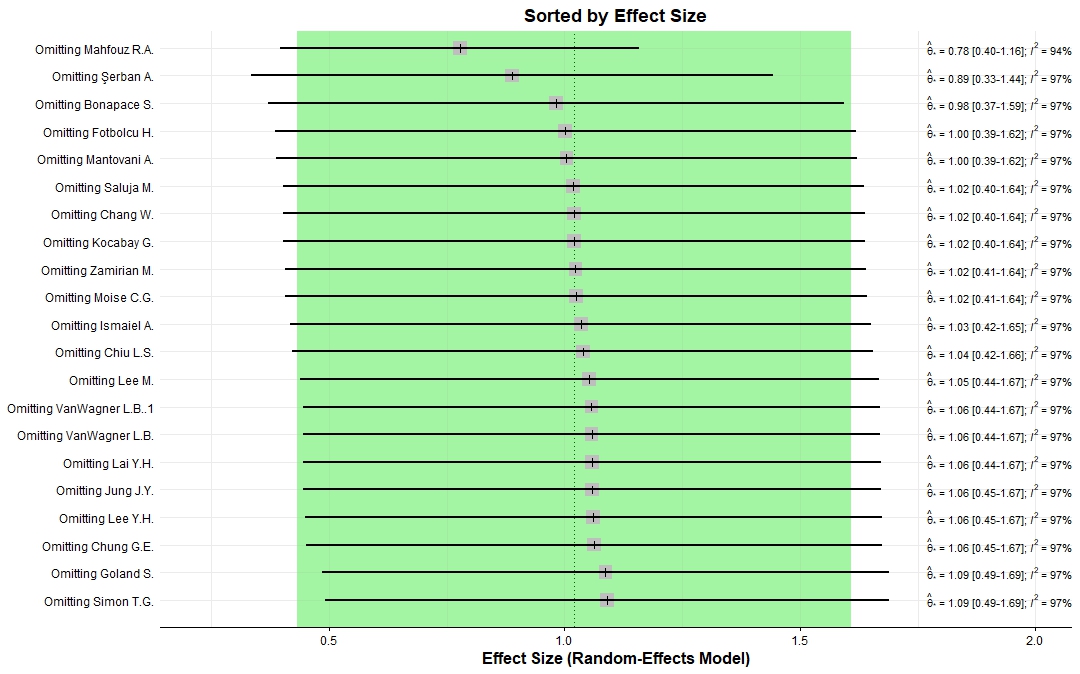

Supplement: Supplementary file 1 [file ijms-24-14292-s001.zip › Supplementary Figure S2.jpeg]

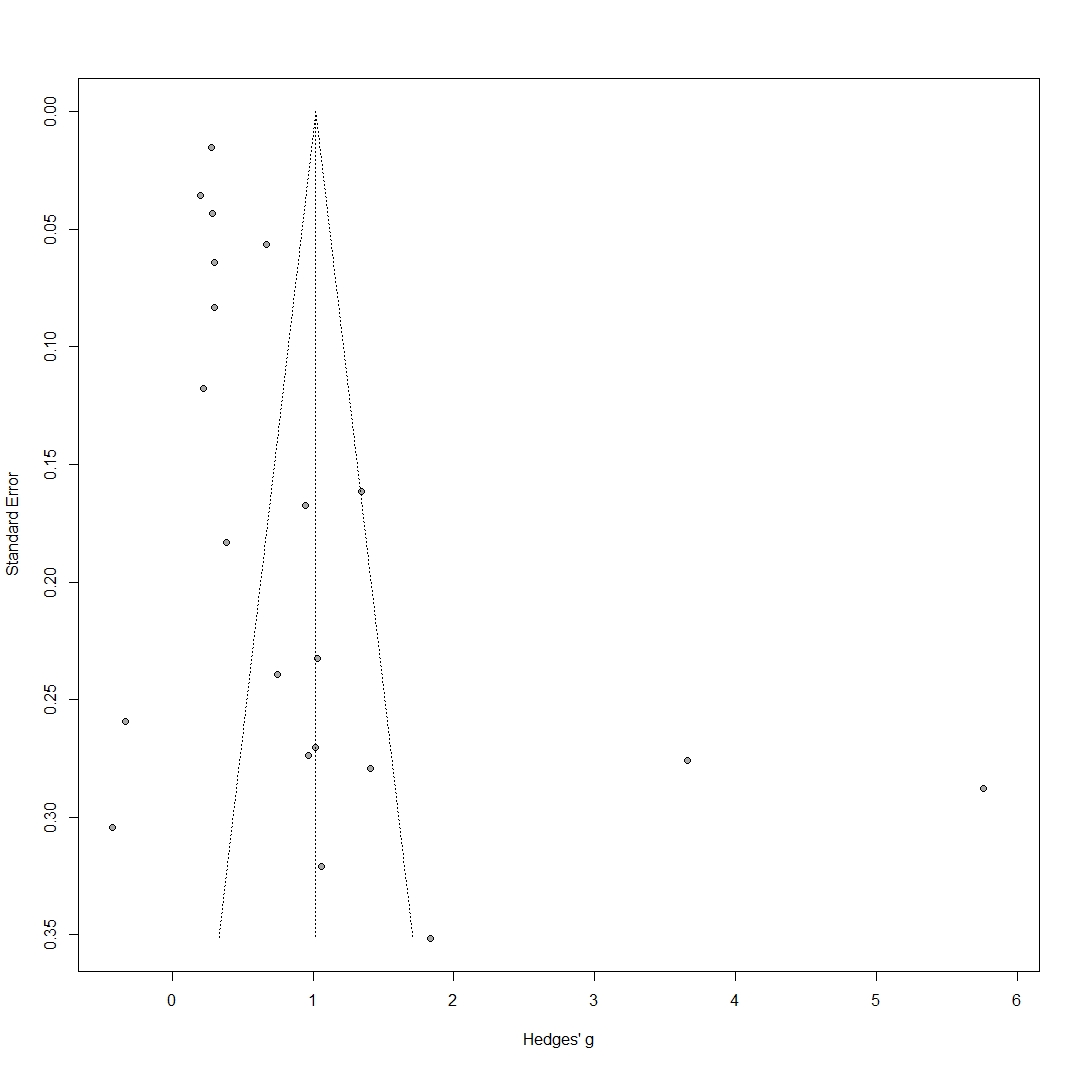

Supplement: Supplementary file 1 [file ijms-24-14292-s001.zip › Supplementary Figure S3.jpeg]

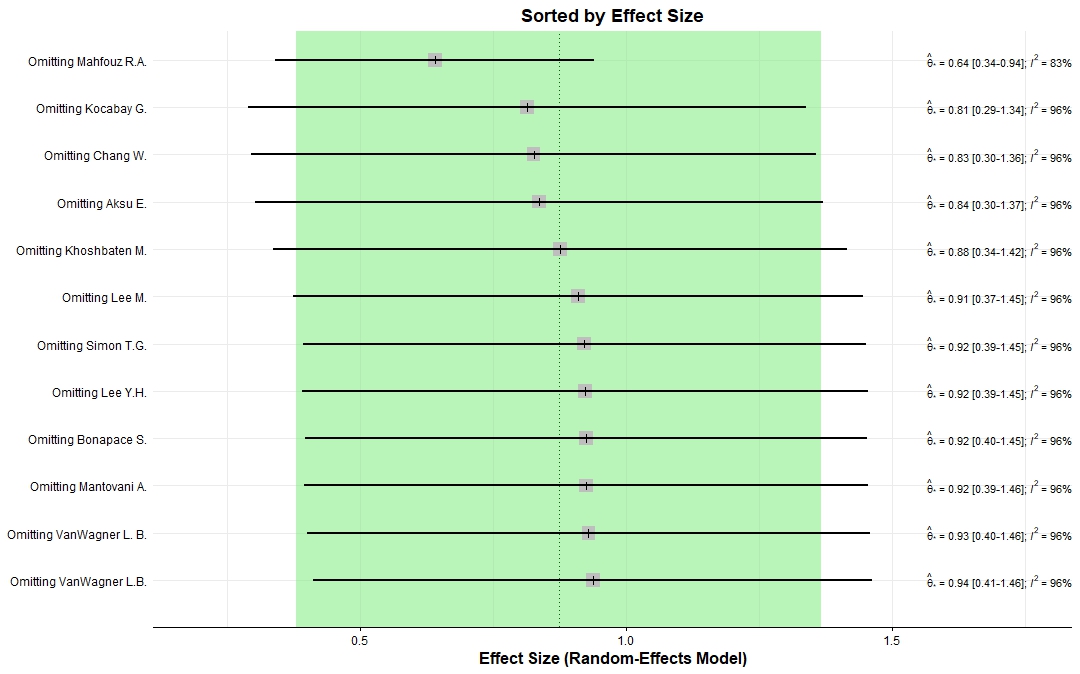

Supplement: Supplementary file 1 [file ijms-24-14292-s001.zip › Supplementary Figure S4.jpeg]

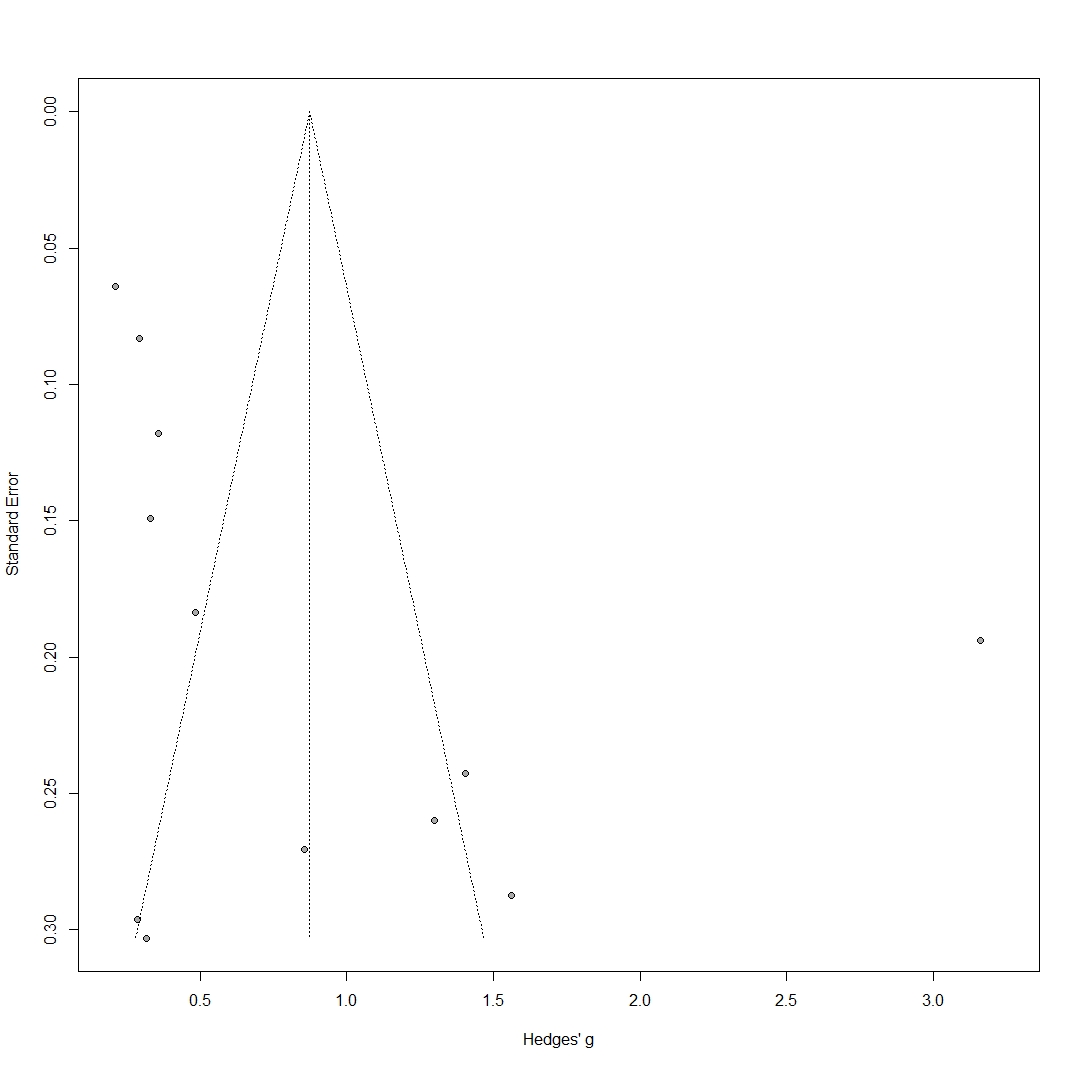

Supplement: Supplementary file 1 [file ijms-24-14292-s001.zip › Supplementary Figure S5.jpeg]

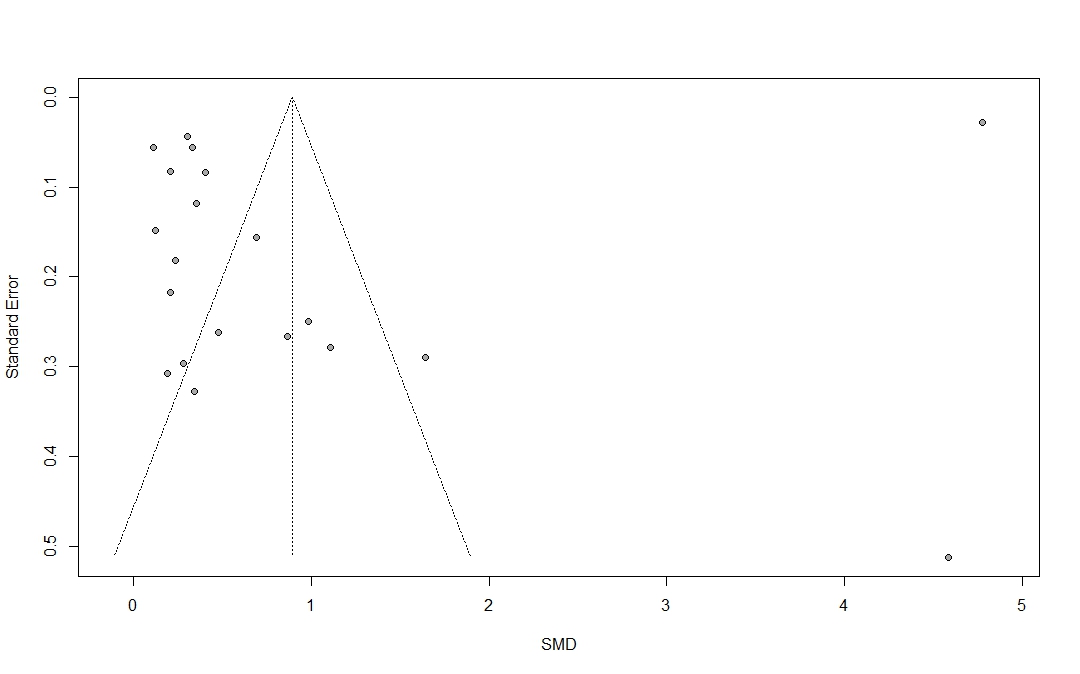

Supplement: Supplementary file 1 [file ijms-24-14292-s001.zip › Supplementary Figure S6.jpeg]
